# Optimizing Prolonged (6 h) Normothermic Machine Perfusion of Donor Kidneys (PROPER Study)

**DOI:** 10.1111/aor.70080

**Published:** 2025-12-31

**Authors:** Asel S. Arykbaeva, Veerle A. Lantinga, L. Leonie van Leeuwen, Marten Engelse, Ton J. Rabelink, Jesper Kers, Jason B. Doppenberg, Volkert A. L. Huurman, Robert A. Pol, Robert C. Minnee, Henri G. D. Leuvenink, Rutger J. Ploeg, Cyril Moers, Dorottya K. de Vries, Ian P. J. Alwayn

**Affiliations:** ^1^ Department of Surgery Leiden University Medical Center (LUMC) Leiden the Netherlands; ^2^ LUMC Transplant Center Leiden the Netherlands; ^3^ Department of Surgery University of Groningen, University Medical Center Groningen (UMCG) Comprehensive Transplant Center Groningen the Netherlands; ^4^ Recanati/Miller Transplantation Institute Icahn School of Medicine at Mount Sinai New York City New York USA; ^5^ Department of Internal Medicine LUMC Leiden the Netherlands; ^6^ Department of Pathology LUMC Leiden the Netherlands; ^7^ Department of Pathology Amsterdam UMC, University of Amsterdam Amsterdam the Netherlands; ^8^ Van't Hoff Institute for Molecular Sciences University of Amsterdam Amsterdam the Netherlands; ^9^ Department of Surgery, Division of Hepatopancreatobiliary and Transplant Surgery Erasmus MC Transplant Institute, University Medical Center Rotterdam Rotterdam the Netherlands; ^10^ Nuffield Department of Surgical Sciences University of Oxford Oxford UK

## Abstract

**Background:**

Ex situ normothermic machine perfusion (NMP) holds great promise in preserving and concomitantly evaluating the viability of kidney grafts. NMP for 1 to 2 h has been shown to be feasible and safe, demonstrating no adverse impact on early graft function. Prolonging the duration of NMP offers an extended timeframe for evaluation, besides creating a window for pretransplant therapeutical interventions. This study aimed to assess the feasibility of extending the duration of perfusion to 6 h.

**Methods:**

We investigated the prerequisites to extend the warm perfusion of donor kidneys safely for up to 6 h. Human donor kidneys deemed unsuitable for transplantation were included for experimental NMP. Throughout the perfusion process, we assessed metabolic activity, as well as the extent of biochemical, hemolytic, and histological injury through biopsy, urine, and perfusate analyses. Stepwise alterations were made to the protocol accordingly.

**Results:**

An analysis of 30 discarded kidneys revealed that improvements in erythrocyte quality, oncotic pressure, and correction of electrolyte imbalances facilitated the achievement of steady flow volumes and ensured a favorable macroscopic appearance of the graft. Extending the perfusion period to 6 h displayed preserved renal viability and stable histological characteristics.

**Conclusions:**

The presented protocol shows prolonging NMP of donor kidneys to 6 h is feasible. We have implemented pivotal elements including the use of fresh (≤ 7 days) washed red blood cells, the addition of albumin, and urine recirculation, resulting in a stable and balanced perfusion. Ongoing refinements are necessary to enable the clinical application of a more prolonged NMP.

## Graphical abstract

Prolonged ex situ normothermic machine perfusion of donor kidneys up to 6 h is feasible, maintaining viability and histological integrity. Key protocol optimizations include fresh red blood cells, albumin supplementation, and urine recirculation—laying the groundwork for assessment and potential pretransplant interventions in future clinical trials.

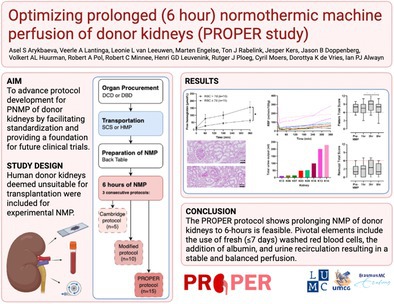

## 1 Introduction

Kidney transplantation is the most effective and cost‐efficient treatment for end‐stage renal disease (ESRD) but remains a limited option due to the scarce availability of donor organs [1]. However, the persistent organ shortage has led to an increased reliance on marginal and extended criteria donor (ECD) kidneys, which are more vulnerable to ischemia–reperfusion injury (IRI), making them prone to developing delayed graft function (DGF) or primary nonfunction (PNF) [1]. Traditional static cold storage (SCS) remains the most widely used preservation method worldwide but offers limited protection against IRI, especially for ECD kidneys. Hypothermic machine perfusion (HMP) has been demonstrated to improve organ preservation compared to SCS and has been embraced in clinical practice [2]. However, HMP operates in a nonphysiological environment, restricting the ability to assess graft viability during preservation [3].

Normothermic machine perfusion (NMP) has emerged as a promising technology for improving kidney preservation by maintaining an organ in a physiologically active state through continuous oxygenated perfusion at body temperature. Unlike SCS and HMP, NMP supports cellular metabolism, enables real‐time viability assessment, and offers the potential for therapeutic interventions [4]. Short‐term end‐ischemic NMP has demonstrated to be safe and feasible, and some studies suggested improved early graft function compared to SCS [5, 6].

This initial success of short perfusion prompted the exploration of prolonged NMP (PNMP). PNMP offers the opportunity to extend preservation times, easing logistics and enabling daytime surgery. More importantly, PNMP may provide a window for therapeutic interventions to initiate kidney repair, such as cell or gene therapy, or targeted pharmacological treatments prior to transplantation, which require longer perfusion times to be effective. Administering these therapies ex situ confines exposure to the donor organ, reducing the risk of systemic side effects in recipients—a significant safety advantage. Investigating PNMP is therefore essential to fully realize its potential to improve both the quality and quantity of donor kidneys available for transplantation.

To date, the longest successful clinical NMP of donor kidney grafts followed by transplantation has been reported by the Toronto group, achieving up to 4.5 h of continuous perfusion, while a recently completed trial by the Oxford group is exploring durations up to 23 h [7, 8]. In preclinical settings, NMP has been sustained for up to 48 h using red‐blood‐cell‐based perfusate with urine recirculation, and in one case, viability was maintained for up to 73 h [9, 10, 11]. However, the limited number of studies, along with variations in methodology, perfusion solutions, and device configurations, has hindered the establishment of PNMP. This variability, scarce step‐by‐step rationale, along with the small number of documented cases, limits insight and thereby deters gathering information to make relevant protocol adjustments for prolonged NMP. This study focuses on developing a clinically safe and feasible protocol for PNMP lasting up to 6 h to be used in the clinical PROPER study prior to transplantation (a safety and feasibility of 6‐h NMP, ClinicalTrials.gov NCT04693325). By addressing practical challenges and optimization strategies and reporting in detail on the methodological approach, this study aims to advance protocol development for PNMP of donor kidneys by facilitating standardization and providing a foundation for future clinical trials.

## 2 Method

### 2.1 Experimental Design

To explore key requirements for 6‐h PNMP, we evaluated three perfusion protocols (Table 1). Initially (*n* = 5), we extended the established 1‐hour NMP protocol from Cambridge [12], at the time the only clinically tested protocol, to 6 h. Due to the Kidney Assist device design (XVIVO, Sweden), we modified the system from closed to open, enabling venous drainage into the reservoir before recirculation.

**TABLE 1:** Machine settings and main components of perfusates used in the three protocols applied in this study.

General	Cambridge protocol	Modified protocol	PROPER protocol
Machine	Kidney Assist	Kidney Assist	Kidney Assist
Pump	Centrifugal	Centrifugal	Centrifugal
Temperature	37°C	37°C	37°C
Pressure (MAP)	Pressure controlled 75 mmHg	Pressure controlled 75 mmHg	Pressure controlled 75 mmHg
Oxygenation	95% O_2_/5% CO_2_ 1 L/min	95% O_2_/5% CO2 0.5 L/min	95% O_2_/5% CO_2_ 0.5 L/min
System	Open	Open	Open
Cold storage solution	UW CSS or MPS	UW CSS or MPS	UW CSS or MPS
First flush	Ringer's solution (200 mL) 4°C	Ringer's lactate (200 mL) room temperature	Ringer's lactate (200 mL) room temperature
Total volume	0.5–0.6 L	0.5 L	1 L
RBCs	1 unit	Washed: 1 unit (~280 mL)	Washed and stored ≤ 7 days: 2 units (~560 mL)
Ringer's solution	250–300 mL	—	—
NaCl 0.9%	—	200–250 mL	400–500 mL
Human serum albumin 20%	—	50 ml	100 ml
Dexamethasone	3.75 mg	—	—
Cefazolin	—	1 g	2 g
Heparin	2000 IU	—	1000 IU
Mannitol 10%	15 mL	10 mL	20 ml
Calcium gluconate 10%	10 mL	10 mL	20 mL
Glucose 5%	—	—	15 mL
Sodium bicarbonate 8.4%	27 mL	10–20 mL	23 mL
Infusions (total pumps)	3	3	3
Epoprostenol (Flolan) 0.5 mg	4 μg/h (venous)	4 μg/h (venous)	8 μg/h (venous)
Nutrient solution	20 mL/h	23.3 mL/h	23.3 mL/h
Amino acids	Synthamin 17 (500 mL)	Aminoplasmal 10%	Aminoplasmal 10%
	Multivitamins (1 vial)	Multivitamins (Cernevit) 0.23 ml/h	Multivitamins (Cernevit) 0.23 mL
	Insulin (100 IU)	—	—
Sodium bicarbonate 8.4%	20 mL/h	—	—
Glucose 5%	5 mL/h	8 ml/h	6 mL/h
Fluid replacement	Urine replacement: Ringer's solution	Urine‐recirculation	Urine‐recirculation

Abbreviations: CO_2_, carbon dioxide; MAP, mean arterial pressure; O_2_, oxygen; RBCs, red blood cells; UW CSS, University of Wisconsin cold storage solution; UW MPS, University of Wisconsin machine perfusion solution.

After observing edema formation, reduced flow from resistance increase, and electrolyte imbalances, we adjusted the protocol. The ‘modified protocol’ (*n* = 10) included human serum albumin (HSA) to raise oncotic pressure [13, 14] and urine recirculation to stabilize electrolytes [11, 15]. Packed RBCs were washed preperfusion to correct baseline electrolytes.

Finally, the ‘PROPER protocol’ (*n* = 15) used washed RBCs stored ≤ 7 days to reduce storage lesions and hemolysis. Perfusate volume was increased to 1 L to prevent air trapping and buffer electrolyte changes during urine output.

The final PROPER protocol perfusion solution was also assessed for 6 h in the absence of an attached donor kidney (*n* = 2). The arterial connection tubing was partially clamped, generating resistance and flow parameters very similar to the standard NMP procedure.

### 2.2 Kidney Procurement and Preparation

**FIGURE 1:**
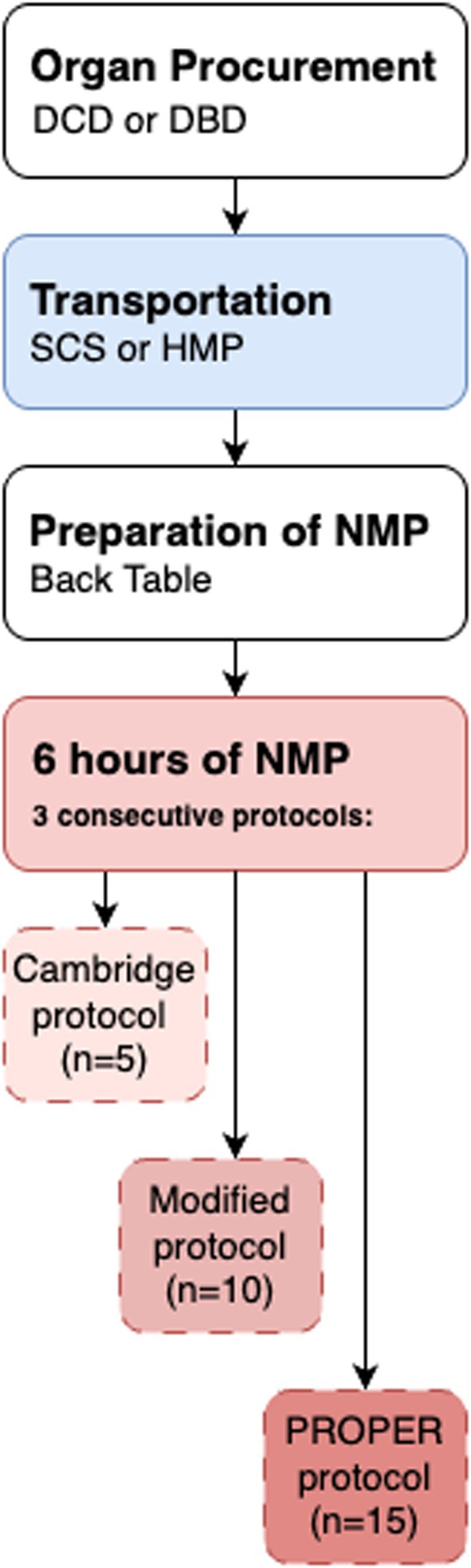
Schematic overview of the study protocol. DBD, donation after brain death; DCD, donation after circulatory death; HMP, hypothermic machine perfusion; NMP, normothermic machine perfusion; SCS, static cold storage. [Color figure can be viewed at wileyonlinelibrary.com]

Kidneys were excluded if they had > 2 arteries or cold ischemia > 24 h. Organs were retrieved after donation after circulatory death (DCD) and donation after brain death (DBD) and were subsequently declined for transplantation due to procurement issues (e.g., malignancy) or quality concerns (e.g., proteinuria) (Table 2). After University of Wisconsin (UW) cold flush, kidneys were preserved via SCS or HMP (Kidney Assist Transport or LifePort Organ Recovery Systems, USA) and sent to LUMC/UMCG for NMP (Figure 1).

**TABLE 2:** Donor characteristics, preservation modality, and ischemic times of kidney grafts (*n* = 30) that underwent 6 h NMP.

Perfusion protocol	Cambridge protocol					Modified protocol									
Kidney ID	C01	C02	C03	C04	C05	M01	M02	M03^a^	M04	M05	M06^b^	M07^a^	M08^b^	M09	M10
Donor age (years)	70	66	63	71	69	71	68	47	67	56	71	47	71	65	54
Donor sex (F/M)	M	M	M	M	M	M	M	M	M	M	M	M	M	M	M
Donor type (DBD/DCD)	DCD	DBD	DCD	DCD	DBD	DCD	DBD	DBD	DCD	DCD	DCD	DBD	DCD	DBD	DBD
BMI (kg/m^2^)	30.6	25.3	26.3	23.7	30.4	23.9	20.7	22.5	24.7	32.7	28.7	22.5	28.7	28.0	27.8
Cause of death	CA	CVA	CVA	CVA	CVA	CVA	CVA	CA	CA	CA	CA	CA	CA	CVA	CVA
WIT (min)	13	13	33	16	0	25	1	0	11	30	11	0	11	0	0
CIT (h)	11.6	19.3	19.4	23.1	27.8	17.9	5.0	5.8	25.6	21.2	2.2	7.5	6.4	17.8	14.5
Initial cold preservation	HMP	HMP	SCS	HMP	HMP	HMP	SCS	SCS	SCS	SCS IGL‐1	SCS	SCS	SCS	HMP	SCS
Left/right kidney	Left	Left	Left	Right	Right	Right	Left	Left	Left	Right	Left	Right	Right	Left	Left
Reason for decline	DCD age and proteinuria	Arterial dissection	Atherosclerosis	Low GFR	Transection in vein	Low GFR	Low GFR	90 min resuscitation of patient	Atherosclerosis	No recipient	Severe kidney failure	90 min resuscitation of patient	Severe kidney failure	Suspected malignancy	Poor perfusion

Perfusion protocol	PROPER protocol														
Kidney ID	K01	K02	K03	K04^c^	K05^c^	K06	K07	K08	K09	K10	K11	K12	K13	K14	K15
Donor age (years)	53	70	63	75	75	54	71	67	66	63	75	76	46	63	52
Donor sex (F/M)	M	M	M	M	M	M	F	F	F	M	F	M	M	M	M
Donor type (DBD/DCD)	DCD	DBD	DCD	DCD	DCD	DBD	DBD	DBD	DCD	DCD	DCD	DCD	DCD	DBD	DCD
BMI (kg/m^2^)	30.6	23.4	35.1	32.2	32.2	24.9	36.8	25.7	24.2	26.3	23.1	24.8	35.9	27.8	31.0
Cause of death	CVA	TC	CA	CVA	CVA	TC	SAB	SAB	CVA	CA	CVA	CVA	TC	SAB	CA
Warm ischemic time (min)	27	0	19	14	14	1	1	0	14	7	15	Not reported	Not reported	0	21
Cold ischemic time (h)	14.3	14.3	16.1	11.4	20.2	22.7	22.1	26.3	23.2	7.1	5.7	8.7	13.4	19.3	13.7
Initial cold preservation	SCS	SCS	HMP	SCS	SCS	HMP	HMP	SCS HTK	HMP	SCS	SCS	HMP	HMP	HMP	HMP
Left/right kidney	Right	Left	Right	Right	Left	Left	Left	Right	Right	Right	Right	Left	Right	Left	Right
Reason for decline	Duodenum perforation	Hepatitis B	Ureter too short	Low GFR	Low GFR	Short renal vein	Atherosclerosis	Artery aneurysm	Artery aneurysm	Low GFR	Proteinuria	Low GFR	Poor perfusion	Short renal vein	Poor perfusion

*Note:* a, b, c paired donor kidneys.

Abbreviations: BMI, body mass index; CA, circulation arrest; CIT, cold ischemia time; CVA, cerebrovascular accident; DBD, donation after brain death; DCD, donation after circulatory death; GFR, glomerular filtration rate; HMP, hypothermic machine perfusion; NMP, normothermic machine perfusion; SAB, subarachnoid bleeding; SCS, static cold storage; TC, trauma capitis; WIT, warm ischemia time.

Upon arrival, kidneys were weighed, inspected, and flushed with Ringer's solution. The renal artery patch was mounted using a holder (XVIVO), and the ureter was cannulated (Ø2.5 mm, Vygon).

### 2.3 Perfusate Composition

The PROPER perfusate contained 2 RBC units (≤ 7 days), washed using an automatic cell‐salvage device (XTRA Autotransfusion System, LivaNova LPC, UK) with 2 L NaCl 0.9%, 100 mL HSA 20%, 400 mL NaCl 0.9%, cefazoline 2 g, calcium gluconate 10% (20 mL), and mannitol 10% (20 mL). Infusions included epoprostenol (4 μg/h), amino acids with multivitamins (23.3 mL/h), and glucose 5% (8 mL/h) (Table 1). pH (7.35–7.45) and glucose (4–7 mmol/L) were maintained with boluses of sodium bicarbonate and glucose (Table S1). Urine was measured and recirculated every 30 min.

### 2.4 Machine Settings

NMP was performed using the Kidney Assist device with pulsatile arterial flow at mean arterial pressure (MAP) 75 mmHg. Perfusate was oxygenated via a membrane oxygenator with carbogen gas (95% O_2_/5% CO_2_) at 0.5–1 L/min.

### 2.5 Kidney Quality Assessment

Kidney quality was assessed macroscopically and by monitoring renal blood flow (RBF), intrarenal resistance (IRR), temperature, and urine output. Hourly arterial/venous blood gases and biochemical analysis were performed using RAPIDPoint (Siemens AG, Germany) or ABL90Flex Plus (Radiometer America Inc., US). Venous perfusate was sampled preinfusion. Samples were centrifuged (1000 *g*) and stored at −80°C. Oxygen consumption was calculated using Fick's principle (Table S2). Perfusate free hemoglobulin (freeHb), lactate dehydrogenase (LDH), and aspartate aminotransferase (ASAT) were measured at the clinical laboratory. Perfusate neutrophil gelatinase‐associated lipocalin (NGAL) and kidney injury molecule‐1 (KIM‐1) were quantified using sandwich enzyme immunosorbent assay (DY1757; DY1750B, Bio‐Techne, USA) following the manufacturer's instructions. Samples from HMP and post‐NMP perfusate were cultured and analyzed in aerobic/anaerobic media for 5–7 days.

### 2.6 Histological and Immunohistochemical Evaluation

Renal biopsies were taken pre‐NMP and after 1, 3, and 6 h using a 4 mm biopsy punch. Biopsies were fixed in formalin, paraffin‐embedded, sectioned (4 μm), and stained with PAS. Remuzzi scores were used to assess chronic injury; Pieters scores for acute tubular injury [16, 17]. A blinded pathologist evaluated all slides.

### 2.7 Statistical Analysis

Data were analyzed using SPSS v23.0 and GraphPad. Parametric data are reported as mean ± SD; nonparametric as median (IQR). One‐way ANOVA tested continuous variable differences. Pearson correlation, paired *t*‐tests, and mixed‐linear models were used for repeated measures.

## 3 Results

### 3.1 Donor Kidney Characteristics

Between May 2018 and December 2022, 30 discarded deceased donor kidneys were included. Majority were procured after DCD (*n* = 18, 60%) and male (*n* = 26, 87%) with a mean age of 64 ± 9 years and BMI of 27.7 ± 4.3 kg/m^2^. Median cold ischemia time was 15h20m (range 2h15m–27h50m). Cold preservation included HMP (*n* = 14, 47%) and SCS (*n* = 16, 53%). Discard reasons included poor function (*n* = 10), vascular anomalies (*n* = 7), donor comorbidities (*n* = 6), retrieval damage (*n* = 4), and perfusion quality (*n* = 3) (Table 2).

### 3.2 Development of Protocol

**FIGURE 2:**
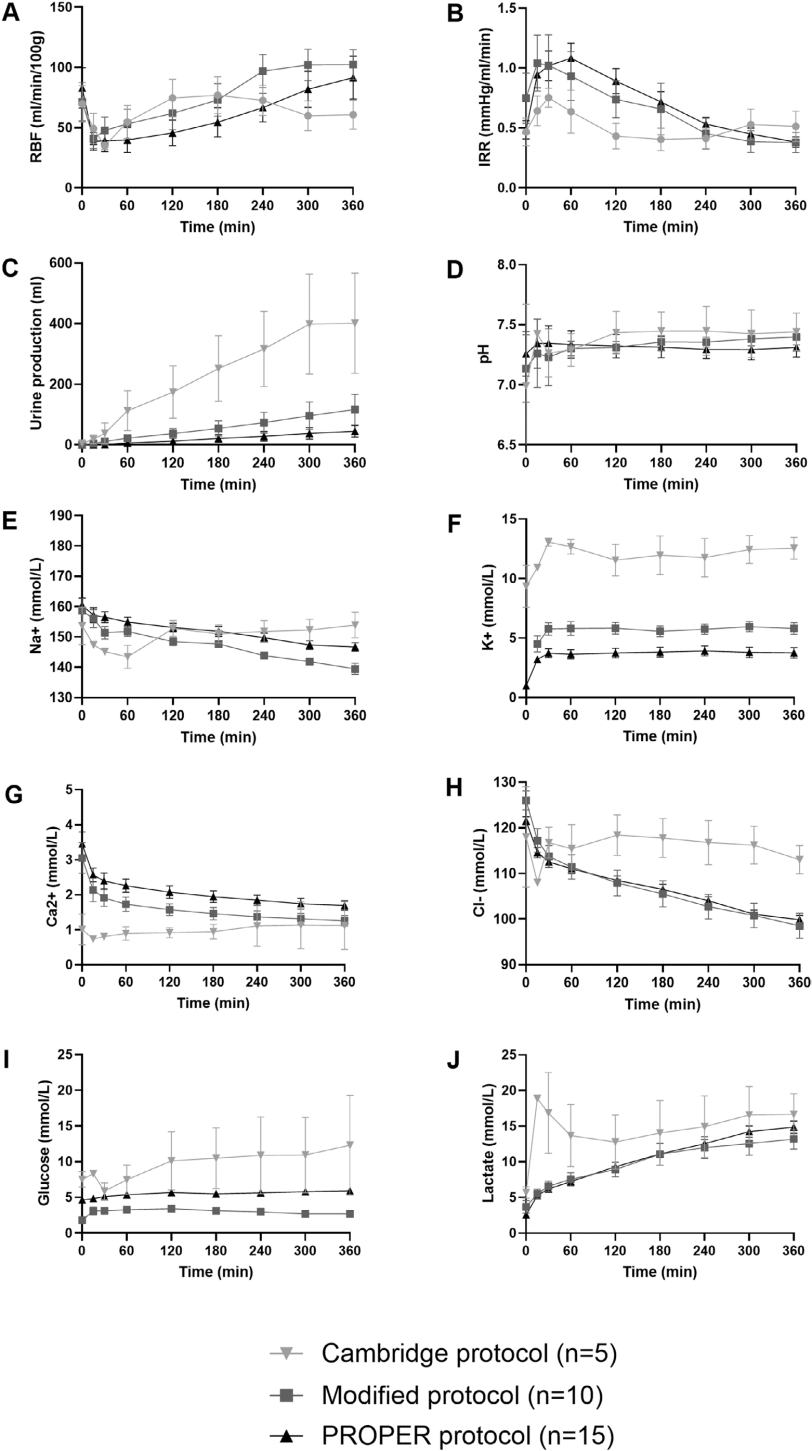
Perfusion and biochemical changes during 6‐h NMP (mean (SD)) shown per study group (Cambridge; Modified; and PROPER protocol). Renal blood flow (RBF) (A), intrarenal resistance (IRR) (B), cumulative urine production (C), pH (D), sodium (Na+) (E), potassium (K+) (F), calcium ion (Ca2+) (G), chloride (Cl‐) (H), glucose (I), and lactate (J) concentration.

Using the initial Cambridge protocol (*n* = 5), RBF declined from 77 ± 34 to 60 ± 24 mL/min/100 g and IRR increased from 0.43 ± 0.24 to 60 ± 24 mmHg/mL/min after 3 h (Figure 2A,B). Electrolyte imbalances and high urine output (401 ± 370 mL) resulted in macroscopic swelling and histological edema and vacuolization (Figure 2C–H, Figure S1).

**FIGURE 3:**
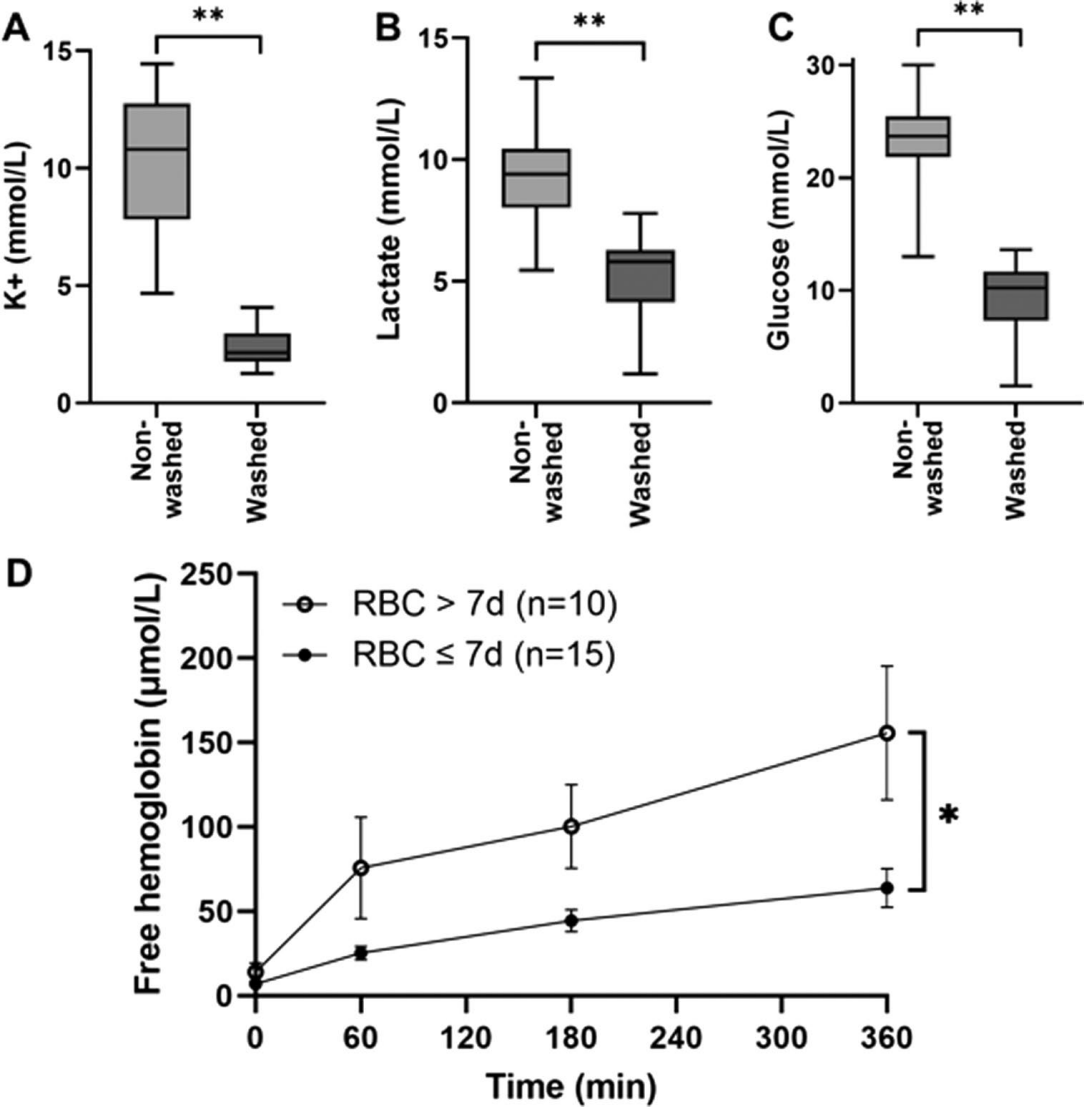
Baseline potassium (K+) (A), lactate (B), and glucose (C) concentrations in banked red blood cells prior to and after the washing process (*n* = 15 mean (SD)). **p < 0.001. Progression of hemolysis (D) during 6‐h NMP using banked red blood cells (RBCs) stored for less than or equal to 7 days, and more than 7 days shown as mean (SEM). *p = 0.006.

Unwashed RBCs resulted in elevated potassium, lactate, and glucose at baseline. After implementing RBC washing using an automatic cell‐salvage device (XTRA Autotransfusion System), this resulted in lower baseline potassium (2.4 ± 0.7 mmol/L), lactate (5.3 ± 1.8 mmol/L), and glucose (9.2 ± 3.3 mmol/L) levels (*p <* 0.001) (Figure 3A–C).

The rapid volume loss due to high urine production resulted in sodium and potassium shifts and fluctuations of the pH. To account for this effect, the urine produced was recirculated and HSA was added to achieve a higher oncotic pressure in the following perfusions (*n* = 10) which improved perfusion stability (Figure 2C–H). However, the weight gain remained, resulting in 10.4% and 13.4% increase in weight in the Cambridge and in the Modified protocol, respectively (Table 3).

**TABLE 3:** Weight increase of kidney graft on NMP (data shown as mean (SD)).

	Prior to NMP (g)	End of 6 h NMP (g)	Difference (%)	*p*
Cambridge protocol	308 (82)	340 (88)	10.4	0.003
Modified protocol	290 (85)	329 (91)	13.4	< 0.001
PROPER protocol	302 (114)	322 (130)	6.6	0.199

Despite the washing step of the RBCs, hemolysis remained a persistent shortcoming when using RBC‐based perfusate. Using fresh, that is, ≤ 7‐day‐old, washed RBCs resulted in a significantly lower degree of hemolysis when compared to older RBCs (*p* = 0.006) (Figure 3D) and a better balance of electrolytes during 6‐h perfusion (Table 3). Perfusate volume was increased to 1 L to prevent air‐trapping and electrolyte fluctuations. The resulting PROPER protocol combined all refinements (Table 2).

### 3.3 Final Perfusion Protocol Outcomes

#### 3.3.1 Hemodynamics

**FIGURE 4:**
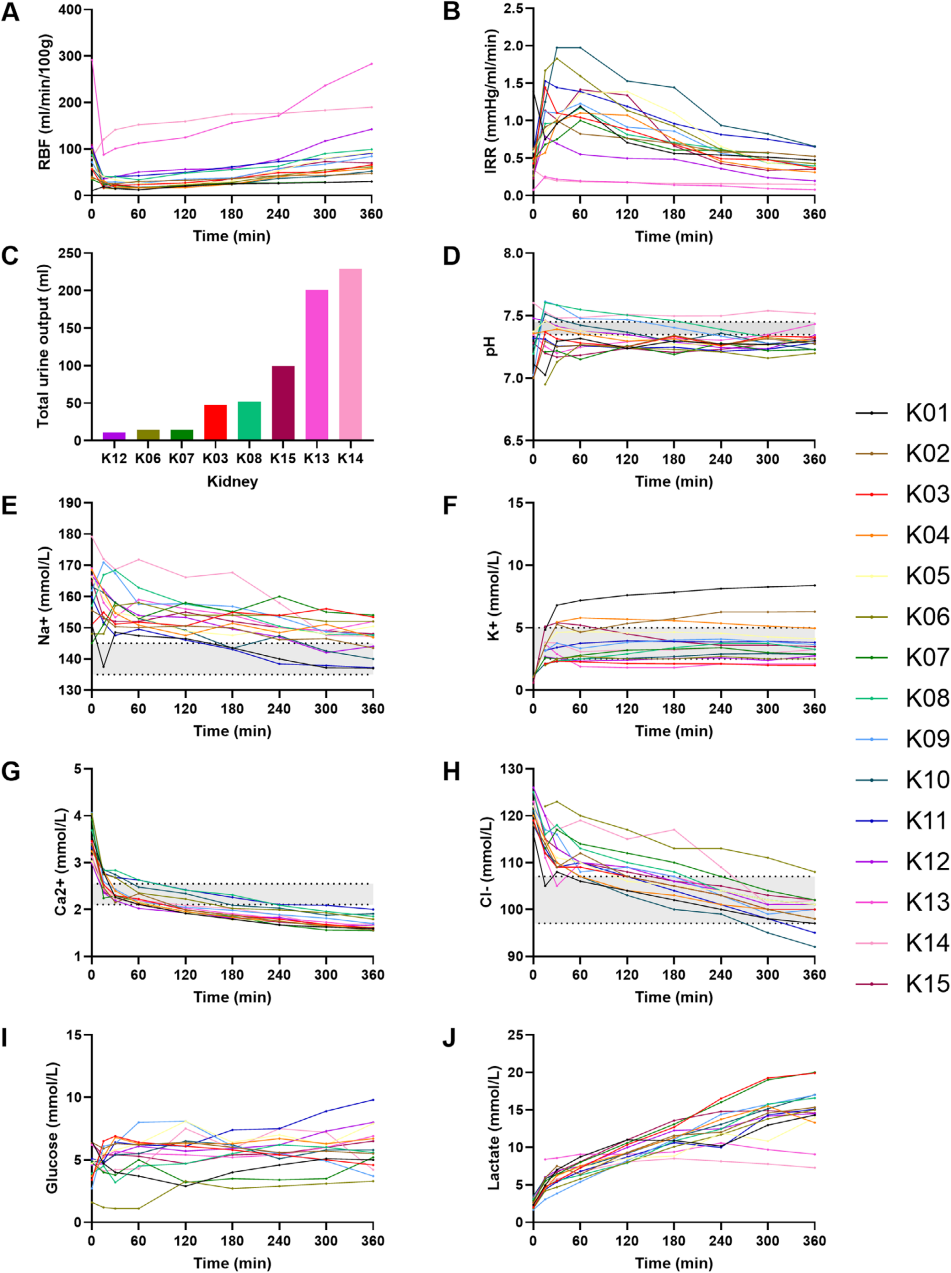
Perfusion and biochemical changes during 6‐h NMP (mean (SD)) shown per kidney graft (*n* = 15) perfused according to the PROPER protocol. Renal blood flow (RBF) (A), intrarenal resistance (IRR) (B), total urine production (C), pH (D); sodium (Na+) (E), potassium (K+) (F), calcium ion (Ca2+) (G), chloride (Cl‐) (H), glucose (I), and lactate (J) concentration. Gray bar = physiological values. [Color figure can be viewed at wileyonlinelibrary.com]

Fifteen kidneys were perfused using the PROPER protocol. Median RBF stabilized at 77 mL/min/100 g (IQR 57–110) and IRR at 0.38 mmHg/mL/min (IQR 0.28–0.49) after 6 h (Figure 4A,B). Eight kidneys produced urine, with a median total urine output of 50 (range 14–176) ml during 6 h of NMP. Urine production correlated moderately with a higher RBF and lower IRR (*p* < 0.001, R^2^ = 0.63) (Figure 4C). HMP‐stored grafts had a significantly higher end flow (116 ± 30 mL/min/100 g vs. 68 ± 10 mL/min/100 g, *p* = 0.026) and urine output (77 ± 32 mL vs. 7 ± 7 mL, *p* = 0.004) compared to SCS‐grafts.

#### 3.3.2 Blood Gas and Biochemical Analyses

After initial shifts during the first 15–30 min, electrolytes stabilized. Sodium declined from 155 (IQR 152–162) to 148 (IQR 144–152) mmol/L (*p* < 0.003), and pH remained within target with minimal bicarbonate use (Figure 4D–H).

**FIGURE 5:**
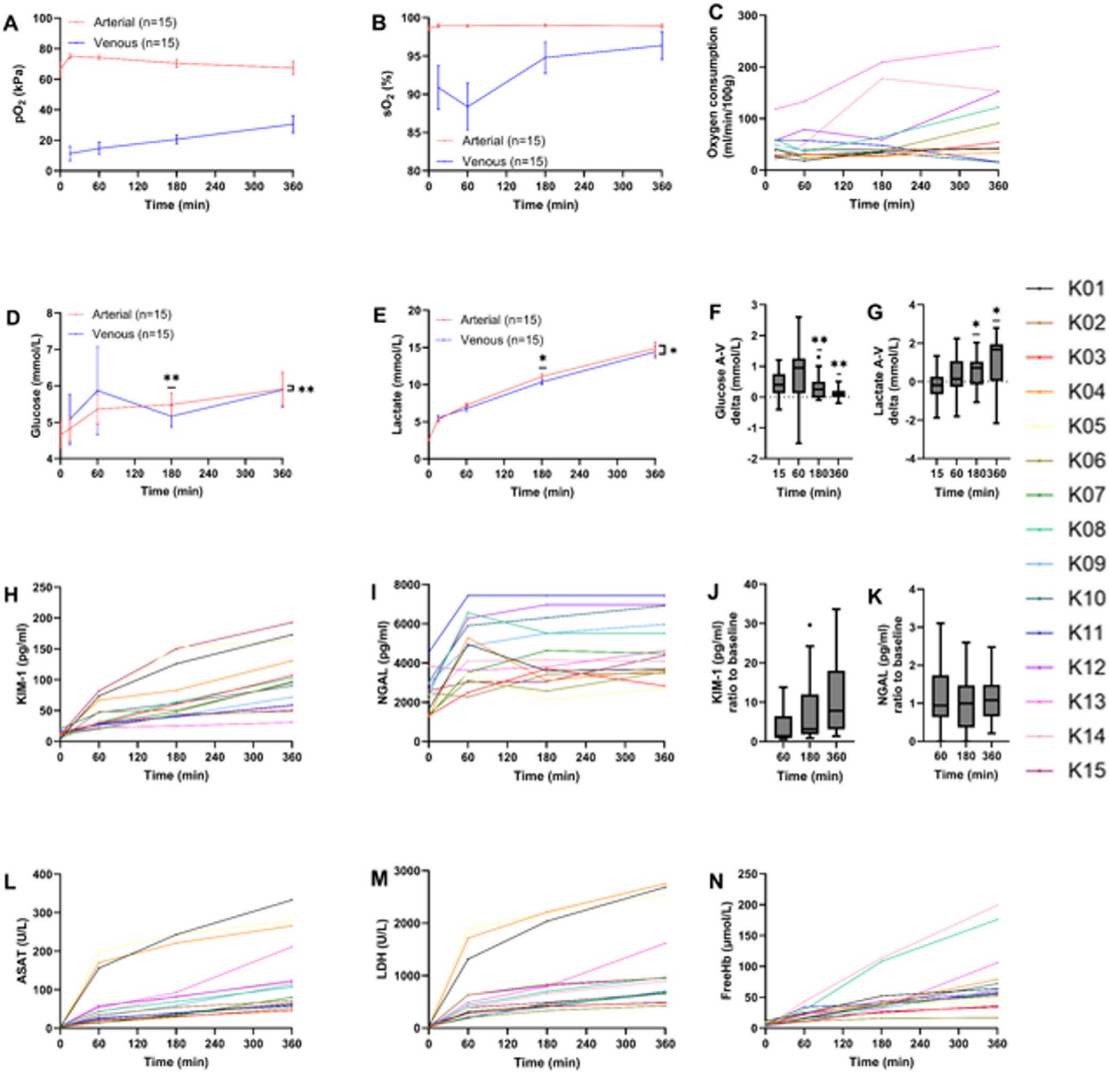
Arteriovenous differences measured in perfusate during 6‐h NMP depicted as mean (SEM; *n* = 15). Oxygen uptake is reflected by pO_2_ (difference range 37–70 kPa) (A) and sO_2_ (difference range 4%–12%) (B), and calculated oxygen consumption (C) (see Table S2 for calculations). Glucose concentration remains constant between 4 and 7 mmol/L, with a continuous uptake by the graft (D, F). Lactate concentrations accumulate in time; however, a continuous lactate uptake by the graft is reflected in the lower concentrations measured in the venous samples (E, G) *p < 0.05, **p < 0.001. Injury markers profile released during 6‐h NMP quantified in perfusate. Kidney injury molecule‐1 (KIM‐1) (H, J) and neutrophil gelatinase‐associated lipocalin (NGAL) (I, K) significant release in perfusate (*p* < 0.001), ratios reflect a continuous release of KIM‐1, whilst NGAL release stagnates. Aspartate aminotransferase (ASAT) (L) and lactate dehydrogenase (LDH) (M) concentrations were significantly different prior to and at the end (6 h) of NMP (*p* < 0.001). Progressively increasing hemolysis was measured by free hemoglobin (free Hb) (*p* < 0.001) (N). [Color figure can be viewed at wileyonlinelibrary.com]

**FIGURE 6:**
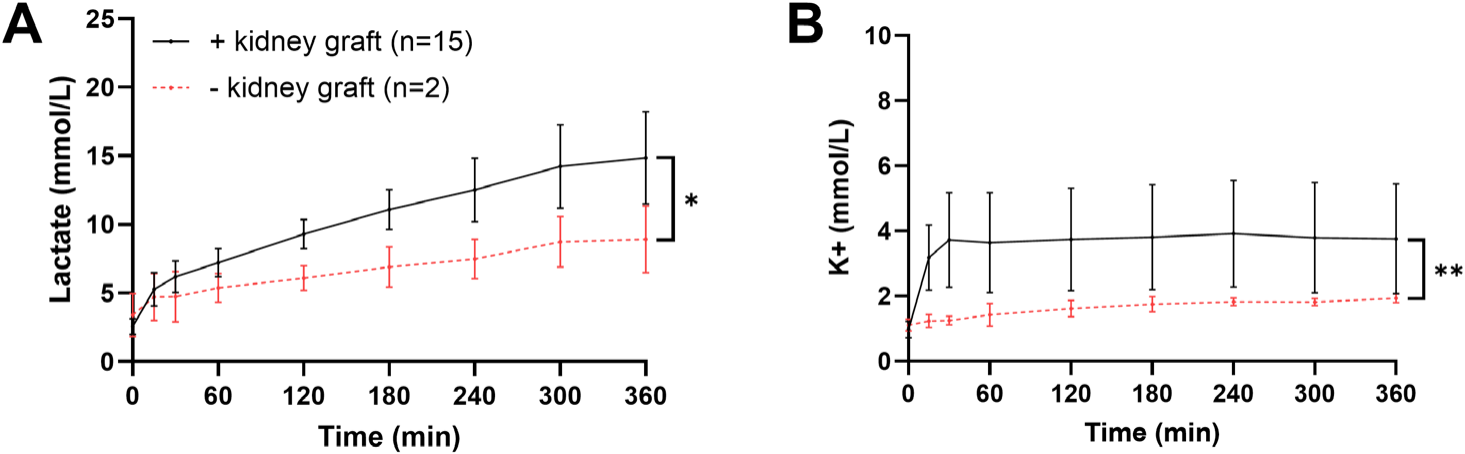
Impact of the NMP system on the perfusate in the absence (red, *n* = 2) and presence (black, *n* = 15) of a kidney graft for (A) lactate and (B) potassium (K+) concentrations depicted as mean (SEM). *p = 0.031, **p < 0.001. [Color figure can be viewed at wileyonlinelibrary.com]

Lactate concentrations significantly increased from 5.2 mmol/L (IQR 4.7–6.0) to 15.0 mmol/L (IQR 14.0–17.0) (*p* < 0.001) in 6 h (Figure 4J). In the setup without a graft (*n* = 2), lactate levels increase delta was less (8.9 mmol/L at 6 h, *p* = 0.031), suggesting that RBCs also contributed to increased lactate production (Figure 6A). From 3 h onwards, a modest yet significant discrepancy between arterial and venous perfusate lactate concentration (*p* = 0.033) was observed, suggesting metabolism of lactate in the kidney (Figure 5E,G).

Glucose was maintained with supplementation and showed significant arterio‐venous consumption (*p* < 0.001) (Figures 4I and 5D,F). Potassium levels remained low in acellular setups (1.9 mmol/L) compared to perfused kidneys (*p* < 0.001) (Figure 6B).

Oxygen consumption remained stable throughout perfusion as shown by the considerable amount of oxygen continuously extracted from the perfusate (Figure 5C). Saturated arterio‐venous difference was smaller (4%–12%) than the renal physiological cut‐off (~20%), compensated by dissolved oxygen extraction (37–70 kPa) (Figure 5A,B).

Furthermore, in the absence of a graft, potassium levels remained low at 1.9 mmol/L and constant compared to perfusions with a kidney (*p* < 0.001) (Figure 6B). The pH and other electrolytes remained comparable to perfusion with grafts (Figure S2).

#### 3.3.3 Injury Markers

ASAT rose from 4 ± 1 to 132 ± 94 U/L; LDH from 24 ± 12 to 1165 ± 826 U/L (both *p* < 0.001). DCD and SCS grafts released more ASAT and LDH than DBD and HMP‐stored grafts (*p* < 0.01) (Figure 5L,M). KIM‐1 increased from 11 ± 5 to 98 ± 49 pg/mL; NGAL from 2434 ± 974 to 4198 ± 1224 pg/mL (both *p* < 0.001), with no significant differences between DCD/DBD grafts. Free Hb rose from 6 ± 3 to 74 ± 51 μmol/L (*p* < 0.001), higher in DBD (100 ± 37) than DCD grafts (61 ± 7 μmol/L, *p* < 0.001) (Figure 5H–K,N).

#### 3.3.4 Microbial Contamination

Of 14 cultures, 3 perfusate cultures (21%) had positive bacterial growth, positive for *Lactobacillus rhamnosus, Staphylococcus warneri*, and *Pseudomonas fluorescens*. All HMP fluid cultures were negative, except for one which had a positive culture (*Streptococcus anginosus (milleri*)) but showed no positive culture at the end of NMP.

#### 3.3.5 Histology

**FIGURE 7:**
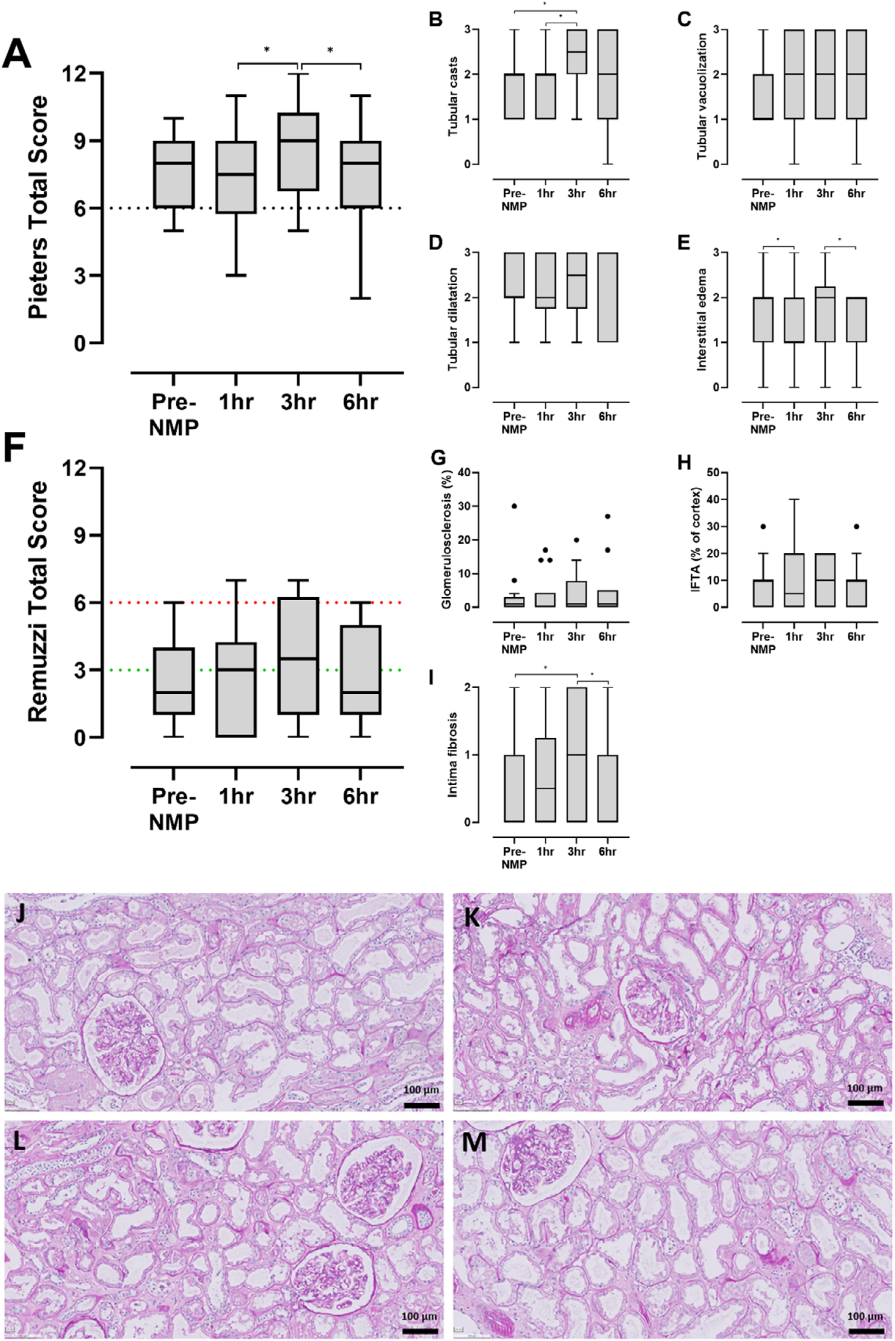
Histological assessment of Period‐Acid Schiff‐stained renal cortex regions of kidneys (*n* = 15) prior to and after 1, 3, and 6 h of normothermic machine perfusion using the PROPER protocol. Composite score for acute tubular injury remained unchanged after 6 h NMP (*p* = 0.702) (A), based on tubular casts (B), vacuolization (C), dilatation (D), and interstitial edema (E) [16]. Fluctuating interstitial edema and tubular casts are reflected over the course of the perfusion. Preexistent chronic damage appeared unchanged (*p* = 0.582) (F), based on glomerulosclerosis (G), interstitial fibrosis and tubular atrophy (H), and arterial narrowing (intima fibrosis) (I) [17]. Green line: 0–3, mild, acceptable for single transplant. Green/red line: 3–6, moderate, acceptable for double transplant. Red line: 6–12, severe, should not be transplanted. *p < 0.05. Example of histological timelapse biopsies collected prior to (J), after 1 (K), 3 (L) after 6 h (M) of normothermic machine perfusion (NMP) using the PROPER protocol (10× magnification). [Color figure can be viewed at wileyonlinelibrary.com]

Tubular injury scores remained unchanged (*p* = 0.702). However, some variation was observed including edema and casts (Figure 7A–E). Preexistent chronic injury remained unchanged during the NMP (*p* = 0.582) (Figure 7F–I) [17]. Overall assessment of pre‐ and end‐NMP biopsies indicated that these grafts, solely based on the histology, could have been transplanted as a single (Remuzzi score 0–3) or double graft (Remuzzi score 3–6). An example of the histological timelapse is shown in Figure 7J–M perfusates.

## 4 Discussion

This study outlines crucial step‐by‐step refinements necessary to enable clinical application of PNMP up to 6 h of donor kidneys prior to transplantation. We show that the clinically established short‐term NMP protocol (1–2 h) [5, 12] cannot be directly extended without substantial modification. Building on prior evidence [12, 13, 18, 19, 20, 21], the addition of HSA and urine recirculation proved beneficial to stabilize flow and electrolyte balance, critical for extended perfusion viability.

A central finding was the effect of RBC storage on perfusion quality. Fresh RBCs (≤ 7 days stored), washed prior to use, yielded in vivo most physiological‐like baseline concentrations of potassium, lactate, and glucose while mitigating hemolysis during NMP. Older RBCs, due to storage lesions, are prone to damage under perfusion conditions [22, 23, 24]. Alternative sources, such as donor‐derived blood or rejuvenation of stored RBCs, may offer further improvements [25, 26].

Initial PNMP attempts resulted in subphysiological perfusion flows and suboptimal temperature control. With modifications, hemodynamic outcomes were more favorable and stable in the PROPER protocol perfused group. The median flow outcomes were comparable to those reported in studies of successfully transplanted grafts by Rijkse et al. after 2 h and Mazilescu et al. after 1 h NMP [5, 7].

Despite a general increase in lactate levels, arteriovenous differences suggested partial renal lactate clearance. However, sustained lactate accumulation in all donor kidneys indicated incomplete metabolic support [27]. This may reflect a state of metabolic imbalance or “normoxic glycolysis,” which, if prolonged, may induce cellular redox stress [9, 11, 21]. Also, this may help assess a kidney's metabolic function and predict recovery of aerobic respiration, distinguishing immediate functioning grafts from DGF grafts [28, 29]. A tailored supplementation of metabolic needs is warranted in future adjustments. Oxygen uptake primarily relied on dissolved oxygen rather than hemoglobin‐bound O_2_, suggesting further optimization of oxygen delivery systems could be explored.

Histologically, renal morphology and tubular injury scores remained stable over 6 h, and chronic damage characteristics of discarded kidneys remained unchanged [30]. Perfusate cultures revealed fewer positive results than the 56% incidence reported in 1 h NMP without antibiotics [31], underscoring the importance of sterility, prophylactic antibiotics, and perfusate culturing to prevent contamination and tailoring post‐transplant recipient treatment.

While graft assessment during NMP is still evolving, it is crucial that NMP itself does not induce additional kidney damage. DCD and SCS‐preserved kidneys (i.e., grafts with more ischemic injury) showed higher ASAT and LDH concentrations at end‐NMP [32]. Kidney‐specific biomarkers KIM‐1 and NGAL were elevated as expected in ischemically injured grafts, although no clear correlation with donor type, preservation method, or ischemia duration was observed.

According to Hosgood's assessment score, twelve (80%) kidneys would be deemed nontransplantable due to absent urine production and low flow in the 1st‐hour [33]—aligning with discarded kidneys, although 20% may merit further evaluation. However, the use of this score is arguable, since a recent randomized controlled trial showed no correlation with DGF [6]. Outcomes of larger cohorts of NMP kidneys will potentially lead to enhanced assessment opportunities and criteria [34, 35, 36]. Additional analyses within the PROPER protocol perfused experiments included extracellular vesicles release, complement activation, and the effect of hemolysis [22, 35, 37].

Discarded human kidneys were used, avoiding animal models due to interspecies differences that could limit clinical applicability. Incorporating albumin and urine recirculation builds on preclinical findings, but some human‐specific factors—like RBC quality—did not appear in pig models using autologous fresh blood. Moreover, metabolic and histologic parameters in marginal grafts differ substantially from animal organs.

A limitation of this study is the inability to measure creatinine clearance, the clinical gold standard for kidney function, as creatinine could not be added in a CE‐approved fashion.

Further improvements in PNMP could include introducing a dialysis membrane for waste removal. Urine recirculation may lead to accumulation of waste products, and persistent increased lactate can lead to intracellular redox stress, worsening the energy crisis [28].

Recent study by Dumbill et al. [38] demonstrated prolonged NMP with a mean duration of 5.83 h, with some up to nearly 24 h and were successfully transplanted. The protocols share many similarities in perfusion medium and settings. The protocol uses one packed cell unit, although its processing is not specified, and applies the same amount of albumin while recirculating urine. Notable strengths include pH and sodium calibration, use of a continuous inline blood gas sensor, and the addition of caspofungin. The perfusion results indicate that more than 2 h are required to reach a steady state. These findings represent a cornerstone for further development, though larger sample sizes and longer follow‐up are needed before defining reliable parameters for assessing kidney quality during NMP.

In conclusion, our study demonstrates that 6‐h renal NMP is feasible with proper protocol modification. RBC washing, albumin supplementation, and urine recirculation formed the foundation for a balanced perfusion. These findings support further clinical evaluation and underscore the importance of harmonized protocols and international collaboration for successful translation of NMP into clinical practice.

## Author Contributions

A.S.A., V.A.L., L.L.L., T.J.R., J.B.D., V.A.L.H., R.A.P., R.C.M., H.G.D.L., R.J.P., C.M., D.K.V., and I.P.J.A. participated in the research design. A.S.A., V.A.L., L.L.L., T.J.R., J.K., J.B.D., V.A.L.H., R.A.P., R.C.M., H.G.D.L., R.J.P., C.M., D.K.V., and I.P.J.A. participated in the writing of the article. A.S.A., V.A.L., L.L.L., J.K., J.B.D., M.E., V.A.L.H., R.A.P., R.J.P., C.M., D.K.V., and I.P.J.A. participated in the performance of the research. A.S.A., V.A.L., L.L.L., J.K., C.M., and D.K.V. participated in data analysis.

## Funding

This work was supported by Nierstichting, BHF1P02.

## Disclosure

The authors have nothing to report.

## Ethics Statement

The study received ethical approval from the Medical Ethical Committees of LUMC and UMCG (B19.019). Consent was obtained from next of kin of deceased donors.

## Conflicts of Interest

The authors declare no conflicts of interest.

## Supporting information


**Data S1:** aor70080‐sup‐0001‐Supinfo.docx.

## Data Availability

The data that support the findings of this study are available from the corresponding author upon reasonable request.
