# Supplementary material for: Optimizing Prolonged (6 h) Normothermic Machine Perfusion of Donor Kidneys (PROPER Study)
Source: Artif Organs. 2025 Dec 31;50(4):550–63. doi: 10.1111/aor.70080 (PMC13125389; doi:10.1111/aor.70080)

# Supplementals

**Supplemental Table 1:** Calculations for glucose concentration and pH corrections.

| **Glucose suppletion (ml glucose 5%) =**  $\frac{(7-{Glucose}_{measured})\times(\frac{V_{perfusate}}{1000})}{0.278}$ |
| --- |
| Glucose target: 7 mmol/L  Glucose measured: measured glucose concentration in mmol/L  V: perfusate volume in mL  Glucose concentration in the 5% glucose: 0.278 mmol/ml |
| **Sodium bicarbonate suppletion (ml NaHCO_3_)=**  $(\left( {10}^{7.3-\left( {pH}_{measured}-\log\left( \frac{\left[ {HCO}_{3 measured} \right]}{0.0307 \times{pCO}_{2 measured}} \right) \right)} \right)\times\left( 0.0307\times{pCO}_{2 measured} \right)-\left( {HCO}_{3 measured} \right))\times(\frac{V_{perfusate}}{1000}))$ |
| pH target = 7.3  pH measured  HCO_3_ measured: measured bicarboncate concentration in mmol/L  pCO_2_ measured: measured partial pressure of CO_2_ in kPa  V: perfusate volume in mL |

**Supplemental Table 2:** Equation of oxygen consumption according to Fick’s principle

| **Oxygen consumption (mlO_2_ * min^-1^  /100 g) =**  $\frac{Hb*0.024794*\left( {sO}_{2 arterial}-{sO}_{2 venous} \right)+K*(pO_{2 arterial}-pO_{2 venous})*Q}{g}*100$ |
| --- |
| g: kidney weight (gram)  Hb: hemoglobin content (mmol/L)  K: solubility constant of oxygen in H_2_O at 37°C (0.0225 mlO_2_ per kPa)  pO_2_: partial oxygen pressure (kPa)  Q: renal blood flow (dL/min)  sO_2_: hemoglobin saturation (%) |

**Supplemental Figure 1**

Example of histological assessment of Periodic acid-Schiff-stained biopsies collected prior to (A) and after 6 hours (B) of normothermic machine perfusion (NMP) using the Cambridge protocol showing increase of interstitial edema (10x magnification).


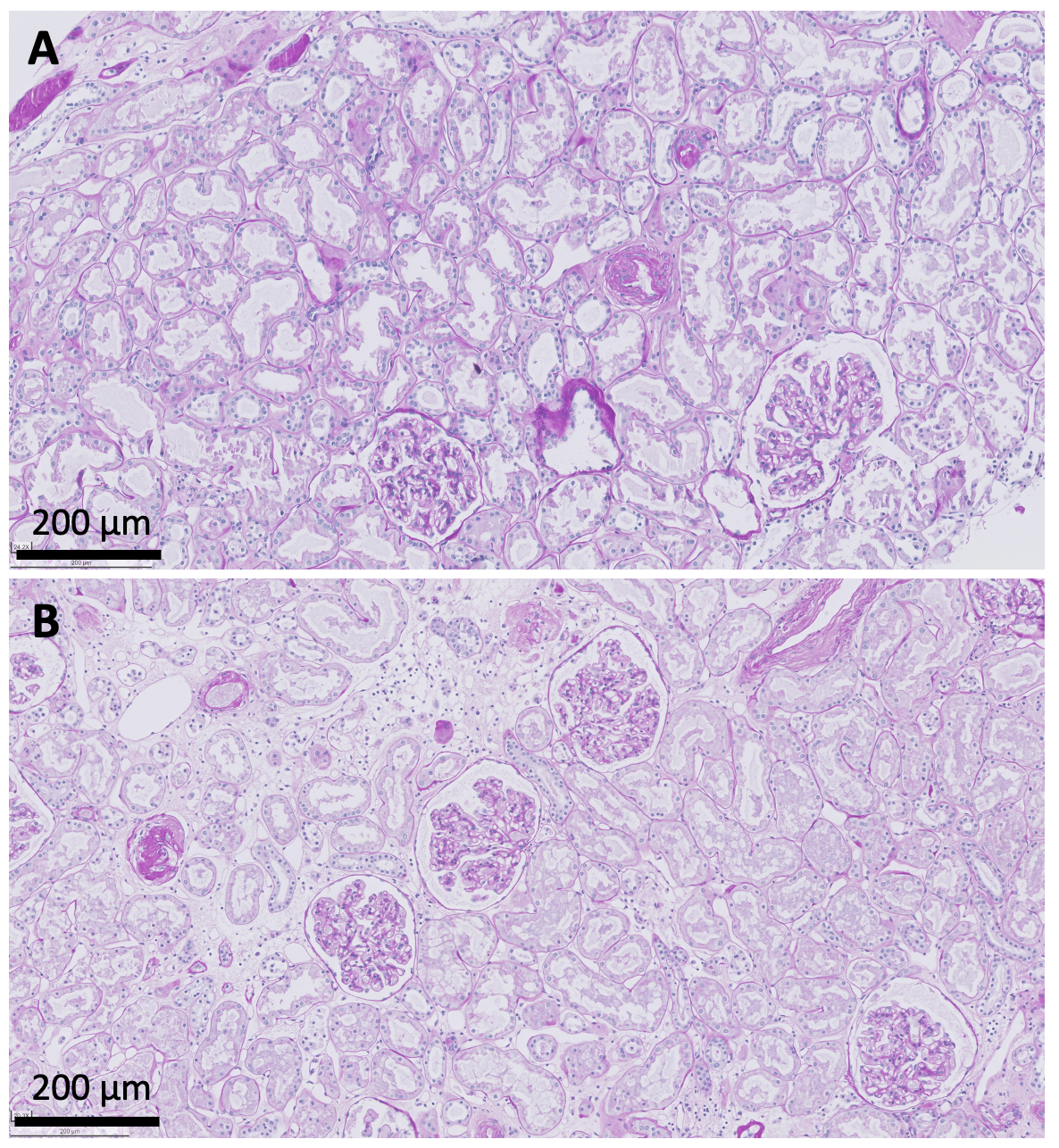


**Supplemental Figure 2**

Impact of the NMP circuit on the perfusate in the absence of a kidney graft (red, n=2) compared to renal perfusions (black, n=15) reflected in (A) pH, (B) glucose, (C) sodium (Na+), (D) calcium ions (Ca2+), (E) chloride (Cl-), depicted as mean (SD). Grey bar: physiological values.


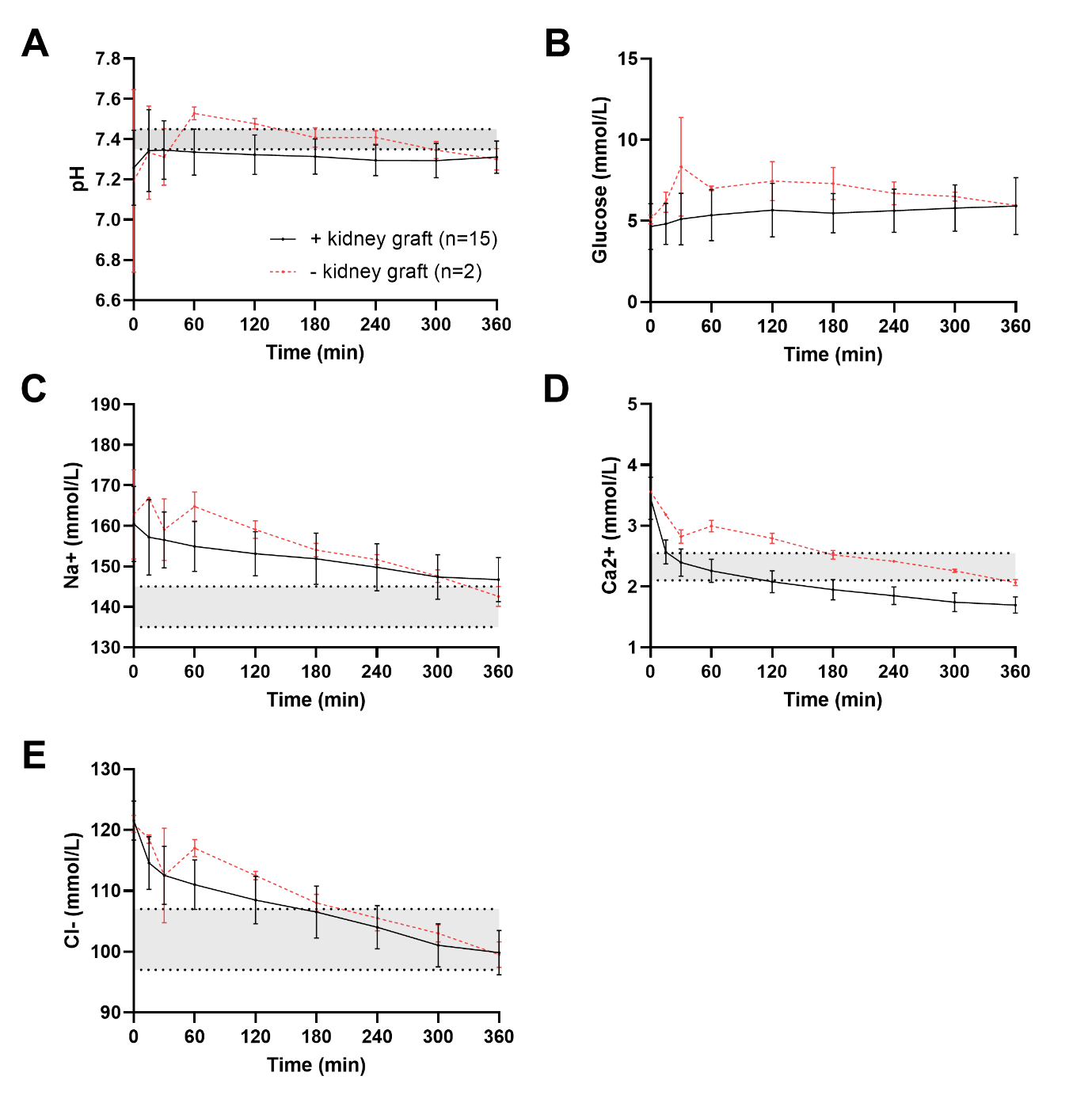

Supplement: Supplementary file 1 — Data S1: aor70080‐sup‐0001‐Supinfo.docx. [file AOR-50-550-s001.docx]
